# Intra-familial Transmission of Hepatitis B virus Infection in Zahedan

**DOI:** 10.5812/ircmj.2282

**Published:** 2013-01-05

**Authors:** Hossein Hatami, Masoud Salehi, Esmail Sanei, Soheila Khosravi, Seyed Moayed Alavian

**Affiliations:** 1 Department of Public Health, School of Health, Shahid Beheshti University of Medical Sciences, Tehran, IR Iran; 2 Infectious Diseases and Tropical Medicine Research Center, Zahedan University of Medical Sciences, Zahedan, IR Iran; 3 Blood Transfusion Research Center, High Institute for Research and Education in Transfusion Medicine, Zahedan Blood Transfusion Center, Zahedan, IR Iran; 4 Department of Internal Medicine, Baqiyatollah Medical University, Tehran Hepatitis Center, Tehran, IR Iran

**Keywords:** Carrier States, Hepatitis B, Transmission, Iran, Prevention and control, HBV Seroprevalence

## Abstract

**Background:**

The household transmission of hepatitis B virus (HBV) is a major health problem. The prevalence rate of this infection is reported about 11% to 57% among family members of HBsAg carriers.

**Objectives:**

This study was conducted to evaluate serological determinants of chronic hepatitis B infection, especially HBsAg positivity, among family members of asymptomatic HBsAg positive carriers in Zahedan (Southeast of Iran).

**Patients and Methods:**

In a cross-sectional study, data were collected from the total number of 454 HBsAg positive cases and 1817 members of their family by trained interviewers and a validated questionnaire. In addition, blood samples were obtained and titrated to detect serologic markers of hepatitis B. All subjects were recruited following informed consent to the study.

**Results:**

In total, 454 chronic HBsAg carriers (66% male) with mean age of 36 ± 10 years and 1817 of their family members were included in the study. The prevalence rate of HBsAg and HBcAb positivity among household members were 19.3% (n = 351) and 51% (n = 573), respectively. The frequency of HBV markers was different by age groups. The highest prevalence rate of HBsAg (34.9%) and HBcAb (31.9%) positivity were found in the age group of 21-30 years old. Importantly, the mothers of index cases had the highest prevalence of HBsAg positivity compared to their spouses who had the lowest proportion (53.2% vs. 8.4%, P < 0.001).

**Conclusions:**

In family members, HBsAg positivity was four times greater than the general indigenous population. Considering the importance of close contacts for transmission, it was more prevalent in mothers of index cases compared to their spouses, suggesting more efficient mother-to-child than sexually transmission of HBV. It was also more prevalent in those having a history of hepatitis B in their maternal family compared to those with paternal one, probably due to more efficient transmission from infected mothers to children. The lower prevalence of HBsAg positivity in lower age groups could be attributed to vaccination of Iranian infants since 1993 and the screening of HBsAg positive mothers during the last two decades.

## 1. Background

Hepatitis B is a viral infection causing both acute and chronic disease. About 2 billion people have been infected and about 350 million people are chronically infected with this virus worldwide. It is endemic in Asia, and most people in the region have been infected with HBV during childhood. In this region, 8% to 10% of adult population are chronically infected (1). One of the main concerns of HBV carriers is transmission of the infection to the family members (2) as the prevalence of transmission of infection in carriers’ family members was 11 to 57%. (3, 4). The risk of hepatitis B infection is determined as 20-60% through the life (5, 6) and the vertical transmission from mother to baby, or child to child plays an important role in intra-familial transmission, whereas in West European and North American countries, the sexual contact is the main mode of transmission (7, 8). It should be considered that if the rate of infection were high, it would lead to higher transmission of disease and also more frequent occurrence of major complications including cirrhosis and hepatocellular carcinoma (9) which is among the first three causes of death from cancer in men, and a major cause of cancer in women in Asia region (1). Furthermore, transmission of the infection during early childhood from mother to infant is accompanied with the risk of chronicity, which causes social and economic burden (10). In literature, the frequency of HBsAg and HBcAb in families has been reported as 11 to 57% and 81%, respectively (3, 4). Many different reported factors were associated with the risk of infection transmission among family members, including sexual contacts, mother-to-infant transmission, contact to body fluids of infected individuals such as skin lesions, usage of common health supplies such as toothbrush and nail clipper, and mucous and skin lesions (3). Currently, there is no accurate information available about prevalence of HBV infection in family members in Sistan-and-Baluchistan province (Southeast of Iran).

## 2. Objectives

Considering the high prevalence of HBsAg positivity in Zahedan, this study was conducted to evaluate serological determinants of chronic hepatitis B infection, especially HBsAg positivity among family members of asymptomatic HBsAg positive carriers in this area in a three-year period from 2008 to 2010.

## 3. Patients and Methods

In the present study, all individuals affected by hepatitis B infection who referred to the Zahedan Hepatitis Control and Treatment Center from 2008 to 2010, as well as their family members were evaluated. After explaining the study and its objectives and obtaining the consent from family members of chronic carriers (HBsAg positivity for more than six months without clinical and/or laboratory evidence of chronic hepatitis), a suitable questionnaire was filled for them. Then, they were referred to laboratory of Blood Transfusion Organization where a 5-mL blood sample was collected from each individual and the tests were performed on the plasma samples using the ELISA method. HBsAg, HBcAb, and HBsAb tests were performed using Dade Behring kit. Validity and reliability of the tests were evaluated according to the regulations and standards of Iranian Blood Transfusion Organization. After collecting the data, the prevalence of HBsAg, HBcAb, and HBsAb was determined in all family members. They were evaluated in two separate groups being either positive or negative for HBeAg. The individuals who were negative for HBeAg and had normal serum enzyme levels and their serum transaminases (AST and ALT) levels were within normal range were considered as passive carriers. On the other hand, individuals with abnormal serum transaminase levels or those with HBV DNA > 105, or individuals with histological evidence or clinical symptoms of cirrhosis, including ascites and esophageal varices, were considered to have chronic hepatitis.

### 3.1. Statistical analysis

Frequency tables including numbers and percentages were produced for demographic characteristics and possible risk factors of the index group. The association of age groups, history of icterus, and family relationship with the presence of HBcAb and HBsAg was assessed using chi-square test. Duration of marriage between HBsAg positive and negative spouses was compared using t-test. All statistical analyses were performed using SPSS software (version 18.0; SPSS Inc. Chicago, Ill, USA) and P-values less than 0.05 was considered as statistically significant.

## 4. Results

According to the results obtained, 454 individuals among those visited Zahedan Hepatitis Control and Treatment Center were positive for HBsAg and considered as the index group; 298 patients (65.6%) were male, and the mean age of them was 36.3 ± 10.5 years (95% CI: 35.3-37.2). Characteristics of the index cases are shown in Table 1. Among the index group, 323 cases (71.1%) were HBsAg positive carriers (66 individuals were HBsAg and HBeAg positive), and 131 patients (28.9%) had chronic hepatitis B. The risk factors of infection are shown in Table 2. Some individuals had several risk factors simultaneously. Moreover, there was a difference between those having the history of hepatitis B in maternal family and those in the paternal one (P < 0.001).

**Table 1:** Demographical Characteristics of the Index Group

Features	Index group (n = 454), No. (%)
**Gender **	
Male	298 (65.6)
Female	156 (34.4)
**Marital status **	
Married	386 (85)
Single	68 (15)
**Occupation**	
Clerk	112(24.7)
Private	160 (35.2)
Household	132( 29.1)
Student	21 (6.4)
Military	29 (4.6)
**Education**	
Illiterate	74 (16.3)
Primary school	98 (21.6)
Below high school diploma	106 (23.3)
High school diploma	105 (23.1)
** Academic**	71 (16.6)

**Table 2:** Possible Risk Factors in the Index Group

Risk factor	Index group (n = 454), No. (%)
**Dental procedures**	151 (33.3)
**Surgery**	79 (17.4)
**Tattoo**	52 (11.5)
**Hospital stay**	12 (2.6)
**Non-IV drug abuser**	22( 4.8)
**Previous blood transfusion**	21 (4.6)
**Extramarital sexual intercourse**	9 (1.9)
**Phlebotomy**	8 (1.7)
**History of hepatitis in maternal family**	230 (54.4)
**History of hepatitis in paternal family**	94 (20.8)
**History of mortality due to hepatitis in family**	110 (24.1)
**Others (stabbing, contact with possibly contaminated cutting objects)**	17 (3.7)

In the present study, 1817 family members (including 94 mothers, 59 fathers, 371 spouses, 924 children, and 369 siblings) were evaluated, among whom 352 individuals (19.5%) were positive for HBsAg. Mothers and spouses with 53.2% and 8.4%, respectively, had the highest and lowest rates of HBsAg positivity (P < 0.001) in the family members.

The prevalence of HBcAb positivity among 1127 family members was 51.1% (n = 576). Mothers and sons with 91.6% and 27.9% (P < 0.001) had the highest and lowest rates of positive HBcAb, respectively. Furthermore, anti-HBs test was positive for 389 (26.53%) out of 1466 HBsAg negative family members before vaccination, and 37 (3.3%) family members were positive only for HBcAb (isolated HBcAb). The results of HBsAg and HBcAb tests for family members are shown in Table 3. Moreover, the frequencies of HBsAg and HBcAb were evaluated in different age groups of the family members. The frequency of HBsAg and HBcAb were different among the age groups (P < 0.001). The highest frequencies were observed in 21-30 years old family contacts (34.9% and 31.7%, respectively). The prevalence of HBsAg positivity in spouses was different (5.5% versus 14.8%; P = 0.003). Duration of marriage was considered as a contributing factor. It was observed that the duration of marriage in the group with HBsAg positive spouses was 16.3 ± 8.5 years, while the duration was 15.3 ± 9.6 years in the group with HBsAg negative spouses, which were not significantly different (P = 0.7). The history of icterus after marriage was also asked and it was found that in the groups with HBsAg positive and negative spouses, 7 (22.6%) and 10 (3.2%) individuals had the history of icterus after marriage, respectively (P < 0.001).

## 5. Discussion

The frequency of hepatitis B in general population varies from 1.7% to 5% in Iran. Although the major mode of transmission is considered to be perinatal transmission and IV drug abuse (11), it is difficult to determine the main route of transmission when this route among family members is not understood. In the present study, the frequency of HBsAg among 1817 family members of HBsAg positive individuals was found as 19.3%, which is not similar to many other Iranian and non-Iranian reports. For instance, the frequency was reported as 22.2% in Babol (North of Iran) (11), 11% in Nahavand, 37.1% in Hamadan (12) (West of Iran) (4), 6% in Golestan province (North-east of Iran (13), 30.5% in Turkey (14), 16.6% in Israel (15), 19.4% in India (16), 21.1% in Brazil (3), and 18.8% in Greece (17). In our study sero-prevalence of infection in family members was almost four times greater than general population of Zahedan. This shows the importance of transmission among family members. Physical contacts among family members can justify the findings. Confirmed presence of HBV DNA in saliva and sweat of individuals infected with HBV shows the possibility of virus transmission through contaminated instruments and cutaneous and non-cutaneous contacts. Higher prevalence of intra-familial transmission of HBV is due to close contacts between family members, mother to child, and sexually transmission among spouses. The difference between the rates of transmission in different studies is unclear and may be due to socio-cultural and habitual states. Conversely, the lower prevalence of HBsAg positivity in lower age groups could be attributed to vaccination of Iranian infants since 1993 and screening of HBsAg positive mothers during the last two decades. Evaluation of the relationship between age and hepatitis indices has been performed in different Iranian and other studies. In a study carried out in Turkey, the highest prevalence of HBsAg and HBcAb were observed in the age groups of 41-50 and 51-60 years old, respectively (14). In another study in Isfahan (Central of Iran), the highest prevalence of HBsAg positivity was reported in the age group of 40-49 years old (18). In other Iranian studies (19), the highest prevalence of HBsAg was reported in the age group of 40 - 49 years old.. Probable causes of age-dependent increasing of transmission can be attributed to lower socioeconomic and heath levels in previous decades, and the possibility of repeated contacts with the virus during the time, as well as risky behaviors such as tattooing among young individuals.

Frequency of HBsAg among different family members was also evaluated. The lower rate of infection in children, sisters, and brothers could be attributed to the national vaccination program, screening of pregnant women for serological indices of HBV, and their lower age (18). Following the infant vaccination program in Saudi Arabia, started in 1989, the rate of HBsAg carriers in children fewer than 12 decreased from 6.7% in 1989 to 0.3% in 1997. After fulfillment of a ten-year public vaccination program in Taiwan, the rate of HBsAg positivity decreased from 9.8% to 1.3%. A study in Iran indicated that after carrying out vaccination program, the prevalence of HBsAg positive in 2-14 years old children and also the rate of vertical transmission after birth decreased significantly (20). Nevertheless, particularly in crowded communities, infected mothers are the main reservoirs of infection leading to horizontal transmission; therefore, cutting the chain of mother-to-infant transmission would remove this important mode of transmission, as well. So, maintaining the infant vaccination program plays an important role to reduce familial transmission of the infection. Re-vaccination of young adults, which is another preventive program in our country, can enhance other preventive measures. A considerable finding of the study was the frequency of 3.3% for isolated HBcAb. The rate was reported as 1.3% in Isfahan (18), 2.5% in Nahavand (4), 2% in Saudi Arabia (21), 10% in Brazil (22), and 13.3% in Egypt (23). The presence of isolated HBcAb (without HBsAg and anti-HBs) can be associated with some clinical conditions, a chronic carrier with undetectable HBsAg serum level, during the window interval between resolution of HBsAg and emergence of anti-HBs, previous infection with undetectable anti-HBs level, and cross-reaction with other antibodies. According to previous studies, HBV DNA was detectable in the serum sample of some of these individuals. For instance, in the study carried out in Zahedan on cases of isolated HBcAb, 2.27% of cases were positive for HBVDNA (24). In different studies in which molecular methods were employed, the rate of HBVDNA in isolated HBcAb was reported as 0.8% in Brazil (22), 10% in Egypt (23), and 12.2% in Shiraz (25), indicating the potential risk of infection transmission from these individuals to other family members. This may be neglected even in primary screening of family members or pregnant women. The prevalence of HBsAg positivity among spouses was 7%. The rate of positive HBsAg was 5.5% in women with infected husbands and 14.8% in men with infected wives (P = 0.003). In a study carried out in Babol, the rate of positive HBsAg was 5.6% in women with infected husband, while it was 22.4% in men with infected wives (P < 0.01) (11). In another study in Turkey, the rate was demonstrated to be higher in men with infected wives than that in women with infected husbands (70% versus 21.9%) (26). The rates were similar in the study carried out in Nahavand (4). The difference in the rate of transmission might be explained by the higher frequency of HBsAg in men, and by differences in the life style. In transmission of the infection to the partners, some factors such as duration of marriage were studied, which was also included in the Babol study (11). Although they mentioned that there was a relationship between duration of marriage and infection of partners, the current study could not find a significant correlation between the mean duration of marriage and the rate of positive HBsAg. In our study, the sero-prevalence of infection in family members was almost four times greater than that in the general population of this area. This shows the importance of close contacts for transmission among family members. The prevalence of HBsAg positivity in mothers of index cases was six times more than that in their spouses, suggesting more efficient mother-to-child transmission compared to sexually transmission of HBV. There was a statistically significant difference between those having a history of hepatitis B in maternal family and those with the paternal one, which may be due to more efficient mothers-to-children transmission compared to fathers-to-children transmission. The lower prevalence of HBsAg positivity in lower age groups could be related to vaccination of Iranian infants since 1993 and the screening of HBsAg positive mothers during the last two decades. Considering the high possibility of hepatitis B transmission among family members, pre-marriage tests, screening of pregnant women, vaccination and training of family members (particularly mothers), pre-marriage consultation for couples one of which is positive for HBsAg, and follow-up HBsAg positive mothers and their infants for receiving prophylactic treatment are important measures to prevent hepatitis B transmission in the community. We must also break the sides and angles of ignorance, poverty, and inequality as an ominous triangle to eradicate hepatitis B and many other infectious diseases.
